# Flux, Impact, and Fate of Halogenated Xenobiotic Compounds in the Gut

**DOI:** 10.3389/fphys.2018.00888

**Published:** 2018-07-10

**Authors:** Siavash Atashgahi, Sudarshan A. Shetty, Hauke Smidt, Willem M. de Vos

**Affiliations:** ^1^Laboratory of Microbiology, Wageningen University and Research, Wageningen, Netherlands; ^2^Research Programme Unit Immunobiology, Department of Bacteriology and Immunology, Helsinki University, Helsinki, Finland

**Keywords:** xenobiotics, halogenated compounds, gut microbiota, xenobiotic-microbiota interaction, dehalogenation genes, metagenomics

## Abstract

Humans and their associated microbiomes are exposed to numerous xenobiotics through drugs, dietary components, personal care products as well as environmental chemicals. Most of the reciprocal interactions between the microbiota and xenobiotics, such as halogenated compounds, occur within the human gut harboring diverse and dense microbial communities. Here, we provide an overview of the flux of halogenated compounds in the environment, and diverse exposure routes of human microbiota to these compounds. Subsequently, we review the impact of halogenated compounds in perturbing the structure and function of gut microbiota and host cells. In turn, cultivation-dependent and metagenomic surveys of dehalogenating genes revealed the potential of the gut microbiota to chemically alter halogenated xenobiotics and impact their fate. Finally, we provide an outlook for future research to draw attention and attract interest to study the bidirectional impact of halogenated and other xenobiotic compounds and the gut microbiota.

## Introduction

The term xenobiotic is usually used in the context of environmental pollutants to refer to synthetic compounds produced in large volumes for industrial, agricultural and domestic use (Atashgahi et al., 2018c). Xenobiotics can enter the environment at high (μg/L to mg/L range) or at “micropollutant” concentrations (ng/L to μg/L range) (Schwarzenbach et al., 2006; Meckenstock et al., 2015). One important group of xenobiotics comprise halogenated compounds with diverse sources and sinks. Halogenated organic compounds, organohalogens, are usually synthesized for industrial, agricultural and pharmaceutical applications (Häggblom and Bossert, 2003). It has also been shown that over 5000 organohalogens are naturally produced from biogenic and geogenic sources (Gribble, 2010). Inorganic halogenated compounds such as chlorine dioxide, hypochlorite, and chlorite are commonly applied as bleaching agents and disinfectants (Liebensteiner et al., 2016). In turn, halogenated compounds can be used as carbon sources, electron donors and acceptors by a diverse array of aerobic and anaerobic microorganisms in growth-dependent and co-metabolic modes (Janssen et al., 2001; Van Pée and Unversucht, 2003; Schneidewind et al., 2014; Peng et al., 2017). As such, microbial degradation represents an important sink of halogenated compounds.

Xenobiotics are also considered as chemical substances from natural or synthetic sources found within an organism that are not naturally produced by the organism or expected to be present. As such, the human body is exposed to variety of (halogenated) xenobiotic compounds, such as persistent organic compounds (POPs), pesticides, pharmaceuticals and personal care products (PPCPs), and food additives. Site-specific microbiomes associated with the gut, skin, or respiratory tract are the first to encounter xenobiotics and mediate “first pass” metabolism prior to compound absorption to internal organ systems (Dietert and Silbergeld, 2015). Among these portals of entry, most interactions between xenobiotics and the human microbiota occur within the human gut (Sousa et al., 2008; Dietert and Silbergeld, 2015). The gut microbiota is a diverse and dense microbial community composed of bacteria, fungi, archaea, and viruses (Li et al., 2014; Nielsen et al., 2014). Its immense metabolic diversity is encoded by the intestinal metagenome, that contains genetic information for multiple xenobiotic detoxification and sequestration functions (Haiser and Turnbaugh, 2013; Spanogiannopoulos et al., 2016). The anoxic environment of the gut is well-suited for a reductive and hydrolytic metabolism. This will generate non-polar low-molecular weight by-products that can be absorbed by the host cells. In contrast, the readily absorbed non-polar xenobiotics are transported and metabolized in the liver by a rich collection of oxidative and conjugative enzymes. Such hepatic metabolism will generate hydrophilic, polar and high-molecular weight metabolites. The latter are secreted via the bile and reach the gut where they can be re-metabolized by reductive and hydrolytic enzymes (Sousa et al., 2008; Claus et al., 2016; Koppel et al., 2017). Thus, xenobiotic metabolism by gut microbiota can exert a profound influence on the toxicity and bioavailability of xenobiotics entering the gut via different routes. The outcome of xenobiotic metabolism may be beneficial (Shin et al., 2013), detrimental or even lethal (Okuda et al., 1998) to the host. In turn, exposure to xenobiotics can alter gut microbiota composition and change metabolic activity (Maurice et al., 2013). This may increase predisposition to various diseases (Wang et al., 2011; Lee et al., 2014; Lu et al., 2015).

Extensive research of the last decades has provided insight into the metabolism of halogenated xenobiotics and opened avenues to harness the metabolic machinery of microbes for bioremediation (Smidt and de Vos, 2004; Sutton et al., 2015; Atashgahi et al., 2017, 2018c; Weatherill et al., 2018). In contrast, much less is known about the flux, impact, and fate of halogenated xenobiotics in host-associated ecosystems like the human gut. As such, there is only very limited information about specific microorganisms, genes, and enzymes responsible for halogenated xenobiotic metabolism in the human gut. This contrasts with the enormous and expanding interest in understanding the role of the gut microbiome in health and disease.

Here, we address the present state of the art on the flux, impact and fate of halogenated compounds in the gut. We first provide an overview of the *flux* of halogenated compounds to highlight their environmental sources and diverse exposure routes of human microbiota to these compounds. Subsequently, we provide an overview of the *impact* of halogenated compounds on the structure and function of the gut microbiota and host cells. Lastly, we review the *fate* and metabolism of halogenated compounds in the gut based on published experimental data and a metagenomic survey of the dehalogenation genes in gut microbiome. In a larger context, we provide a rationale for studying the bidirectional impact of (halogenated) xenobiotic compounds and the gut microbiota, i.e., toxicant-microbiota interactions.

## Flux

**Figure 1:**
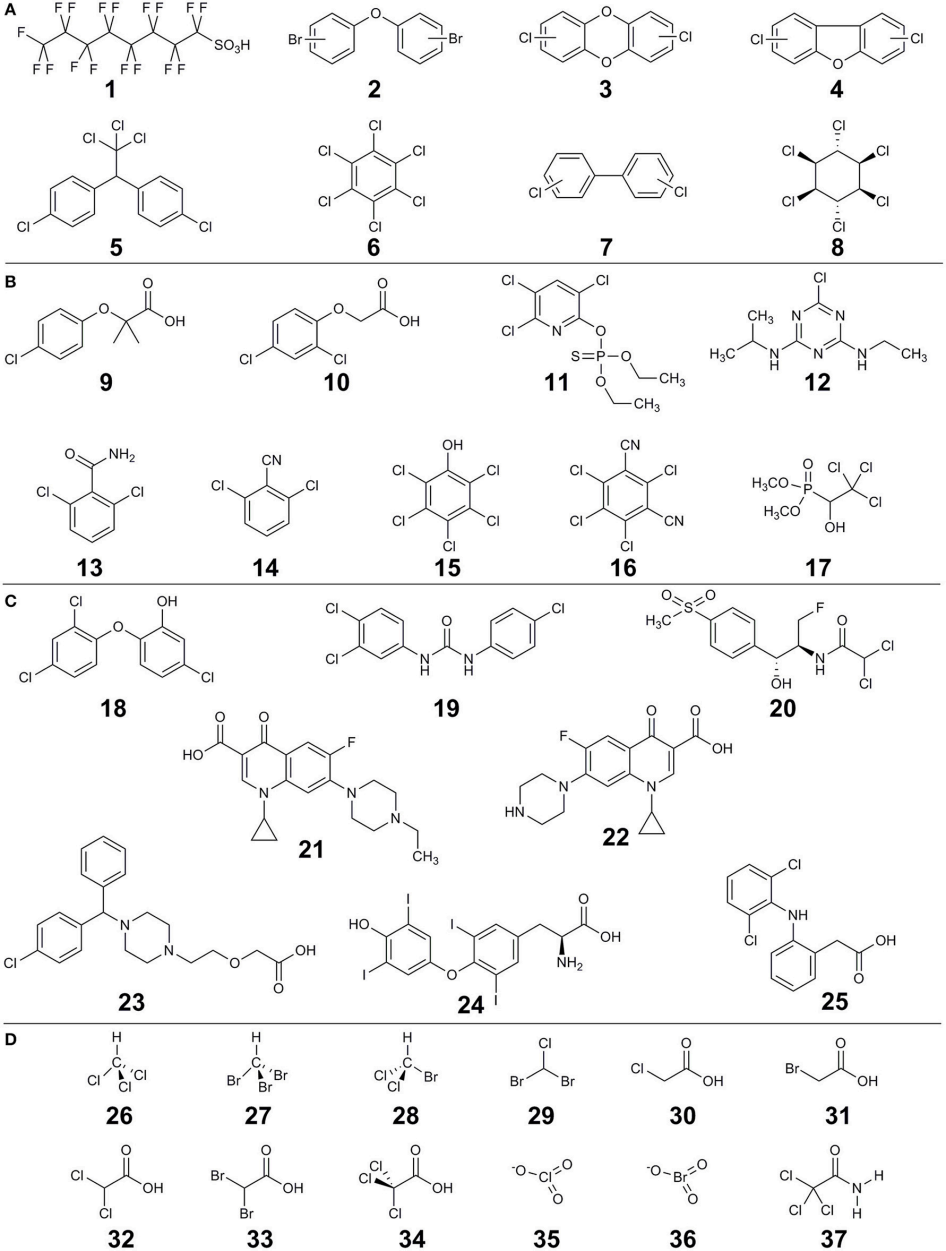
Examples of halogenated compounds discussed in this review. **(A)** persistent organic compounds (POPs), **(B)** pesticides, **(C)** pharmaceuticals and personal care products (PPCP) and **(D)** disinfection by-products (DBPs). Compounds are: perfluorooctanesulfonic acid (**1**), polybrominated diphenyl ethers (**2**), polychlorinated dibenzo-*p*-dioxins (**3**), polychlorinated dibenzofurans (**4**), dichlorodiphenyltrichloroethane (**5**), hexachlorobenzene (**6**), polychlorinated biphenyls (**7**), γ-hexachlorocyclohexane (lindane) (**8**), clofibric acid (**9**), 2,4-dichlorophenoxyacetic acid (**10**), chlorpyrifos (**11**), atrazine (**12**), 2,6-dichlorobenzamide (**13**), 2,6-dichlorobenzonitrile (**14**), pentachlorophenol (**15**), chlorothalonil (**16**), trichlorfon (**17**), triclosan (**18**), triclocarbon (**19**), florfenicol (**20**), enrofloxacin (**21**), ciprofloxacin (**22**), cetirizine (**23**), thyroxine (L) (**24**), diclofenac (**25**), chloroform (**26**), bromoform (**27**), bromodichloromethane (**28**), chlorodibromomethane (**29**), chloroacetic acid (**30**), bromoacetic acid (**31**), dichloroacetic acid (**32**), dibromoacetic acid (**33**), trichloroacetic acid (**34**), chlorate (**35**), bromate (**36**), trichloroacetamide (**37**).

**Figure 2:**
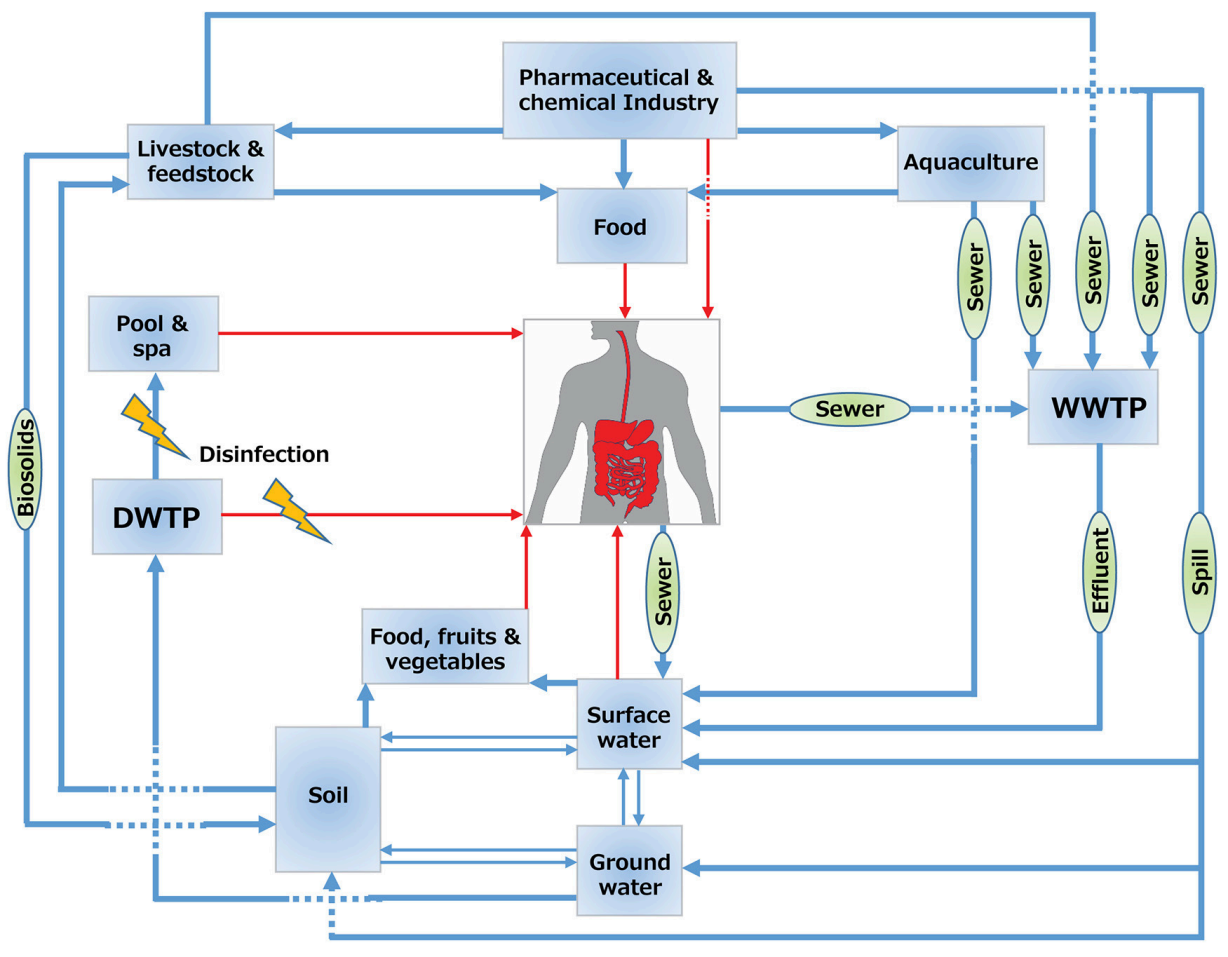
Flux of halogenated compounds. Blue arrows show the flux in the environment and red arrows show the flux to humans. WWTP, wastewater treatment plant; DWTP, drinking water treatment plant.

The main sources of halogenated (micro)pollutants are from industry (e.g., POPs), agriculture (e.g., pesticides), domestic use (e.g., PPCPs) and disinfection by-products (DBPs). Some examples of the halogenated (micro)pollutants that are discussed in this review are shown in Figure 1. Humans come into contact with halogenated xenobiotics through: (i) oral exposure (eventually ending up in the gut), (ii) inhalation (via nose and lungs), (iii) dermal exposure (through the skin), and (iv) ocular exposure (through the eyes) (Figure 2). Oral ingestion is the main exposure route of the general population to halogenated xenobiotics. This is especially the case for the chronic exposure to micropollutant concentrations of residues in food, vegetables, fruits, and drinking water (Boxall et al., 2006, 2012; Damalas and Eleftherohorinos, 2011). In contrast, dermal/inhalation exposure due to showering, bathing, and swimming through daily-life and/or recreational activities is a more important route of exposure to DBPs than oral exposure (Villanueva et al., 2006a,b). Inhalation, dermal and ocular routes are more relevant to occupational exposure of workers in- or nearby residents of- industrial production plants, farms or greenhouses that produce and use halogenated xenobiotics (Damalas and Eleftherohorinos, 2011; Besis and Samara, 2012).

Raw sewer and treated effluents from wastewater treatment plants play a key role in the spread of halogenated compounds in the water cycle (Figure 2). Most current waste- and drinking water treatment plants are not (optimally) designed for the removal of halogenated compounds or their transformation products (Heidler and Halden, 2009; Noguera-Oviedo and Aga, 2016). The latter is particularly relevant as sometimes the transformation products can be more toxic than the parent compounds. For example, perfluorooctanesulfonic acid (compound number **1** in Figure 1) is a POP that can also be produced through biotransformation of other synthetic chemicals such as perfluoroalkyl acids in wastewater treatment plants (Guerra et al., 2014). Similarly, biodegradation of clofibric acid (**9**) leads to production of the more toxic 4-chlorophenol (Salgado et al., 2012). Trace concentrations of such contaminants of emerging concern may eventually end up in finished drinking water due to their toxicity and persistence (Benotti et al., 2008).

Biosolids from the treated sewage sludge represent concentrated sources of hydrophobic organohalogens such as the flame retardant polybrominated diphenyl ethers (**2**), and the biocidal compounds triclosan (**18**) and triclocarbon (**19**). These compounds can desorb and contaminate soil and water, once biosolids are applied as fertilizer (Andrade et al., 2015) (Figure 2). Similarly, organohalogen antibiotics such as fluoroquinolones added to animal feed for disease prevention or for growth promotion may end up in biosolids from livestock production (Martínez-Carballo et al., 2007). Once applied as manure for fertilization of arable land, these compounds can reach humans via different routes. For example, veterinary organohalogen drugs such as florfenicol (**20**) and enrofloxacin (**21**) can be taken up by plants such as lettuce (Boxall et al., 2006).

Recent non-targeted analysis has further highlighted the environmental footprint of organohalogens. For example, monitoring of bottlenose dolphins has identified 327 organohalogens of synthetic as well as natural origins (Shaul et al., 2015). This indicates severe bioaccumulation of organohalogens in marine food webs. A similar non-targeted screening of the sediments of Lake Michigan has identified 1,593 organobromine compounds, many of which were not known previously (Peng et al., 2015). The deposited organohalogens in sediments can be chronically released (Yamashita et al., 2000) and bioaccumulate in organisms in food webs and thereby reach humans.

### POPs

POPs are long lived organic compounds that resist biological, chemical, and photolytic degradation. They exhibit high lipid solubility and hence bioaccumulate in fatty tissues and become concentrated as they move up the food chain. POPs are dominated by organohalogens such as polychlorinated dibenzo-*p*-dioxins and furans (**3**, **4**), dichlorodiphenyltrichloroethane (**5**), hexachlorobenzene (**6**), polychlorinated biphenyls (**7**), γ-hexachlorocyclohexane (lindane, **8**), perfluorooctanesulfonic acid, polybrominated diphenyl ethers, etc.

Although application of organochlorine pesticides and polychlorinated biphenyls has been restricted since the late 1970s, they are still among the most ubiquitous and concerning environmental pollutants due to their persistence, toxicity and bioaccumulation (Xu et al., 2017). Moreover, electronic waste (e-waste) continues to produce POPs such as polychlorinated dibenzo-*p*-dioxins and furans, polybrominated diphenyl ethers, and polychlorinated biphenyls. The e-waste recycling industry in developing countries has drawn the world's attention as a new source of environmental contamination by POPs (Leung et al., 2007; Zhang et al., 2010). POPs can also be produced naturally. For example, polychlorinated dibenzo-*p*-dioxins and furans are formed as by-products of high-temperature processes, such as volcano outbursts, forest fires and waste incineration. The endocrine-disrupting flame retardant polybrominated diphenyl ethers have natural counterparts that are produced by marine sponges (Agarwal et al., 2017) at levels that can exceed 10% of the sponge tissue dry weight (Unson et al., 1994). Naturally produced organohalogens were reported to bioaccumulate in marine mammals (Vetter et al., 2002; Teuten and Reddy, 2007) and humans (Wan et al., 2010; Wang et al., 2012) indicating human contact through the marine food web. Accordingly, POPs can come into contact with humans primarily through dietary intake including fatty fish, red meat and poultry (Kiviranta et al., 2004; Schecter et al., 2010), but also through inhalation and dermal absorption (Besis and Samara, 2012).

### Pesticides

Pesticides such as herbicides, insecticides, and fungicides have been used since the 1940s for agricultural and non-agricultural purposes (Schwarzenbach et al., 2010). Chlorinated phenoxy acid herbicides such as 2,4-dichlorophenoxyacetic acid (**10**), the broad-spectrum chlorinated organophosphate chlorpyrifos (**11**) and the herbicide atrazine (**12**) are among the most intensively used pesticides worldwide (Bradberry et al., 2000; Arias-Estévez et al., 2008; John and Shaike, 2015). Pesticide contamination from specific point sources (μg/L to mg/L range) can be due to accidental releases at manufacturing plants, spills on farm yards and from wastewater treatment plant effluent (Vandermaesen et al., 2016). In contrast, diffuse contamination (ng/L to μg/L range) originates from actual pesticide application that results in large-scale contamination of groundwater through leaching, and contamination of surface water through runoff, erosion, drainage, and drifting (Holvoet et al., 2007). Moreover, contamination can be due to transformation products. For example, 2,6-dichlorobenzamide (**13**) is a highly mobile and persistent groundwater pollutant that originates from transformation of the widely used herbicide dichlobenil (2,6-dichlorobenzonitrile) (**14**) (Horemans et al., 2017). Besides oral ingestion as the main route of exposure, human contact with pesticides could be through: (i) inhalation by breathing the mobile pesticides e.g., during on farm pesticide spraying, (ii) dermal, and (iii) ocular routs e.g., during accidental splashing or spraying pesticides on unprotected skin/eyes of agricultural workers and workers in the pesticide industry (Damalas and Eleftherohorinos, 2011).

### PPCPs

Pharmaceuticals are used to treat or prevent disease or as feed additives in animal farming, whereas personal care products are used in personal hygiene and for beautification, and include products such as shampoos, toothpastes, moisturizers, deodorants, lipsticks, perfumes, etc (Boxall et al., 2012). Whereas the main route of PPCPs release into environment is generally excretion to the sewage system following use, manufacturing facilities can be important local point sources (Ebele et al., 2017) (Figure 2). For example, effluent from a wastewater treatment plant of a major drug manufacturer contained ciprofloxacin (**22**) at a concentration of up to 31 mg/L, that exceeds levels toxic to some bacteria by over 1000-fold (Larsson et al., 2007). A subsequent study found ciprofloxacin (up to 6.5 mg/L) and cetirizine (**23**) (up to 1.2 mg/L) in two lakes in the same region impacted by the wastewater treatment plant effluent (Fick et al., 2009). These mg/L concentrations are 100,000 to 1 million times higher than reported levels of fluoroquinolones in surface waters contaminated by effluents from wastewater treatment plants (Kolpin et al., 2002; Xiao et al., 2008).

Triclosan and triclocarban are broad-spectrum phenolic organochlorine biocides with activity against both bacteria and fungi. These chemicals are found in a wide variety of consumer products, including soaps, detergents, toothpaste, medical devices, plastics, and textiles (Pycke et al., 2014). As a result of common and widespread use, humans are exposed to these chemicals via different routes including absorption (e.g., soaps, toothpaste), ingestion (e.g., drinking water, food), inhalation (e.g., aerosols, dust), and injection/implantation (e.g., medical sutures and devices) (Halden, 2016). For example, according to a survey in 2003-2004, triclosan was found in about three-quarters of urine samples analyzed in the USA at concentrations of 2.4–3790 μg/L (Calafat et al., 2008). A later analysis of triclosan, triclocarban, thier metabolites and by-products in maternal urine and cord plasma in an urban population in the USA has shown widespread fetal exposure to these compounds (Pycke et al., 2014). Triclosan and triclocarban have been detected in aquatic environments such as groundwater, drinking water, wastewater, sewage sludge, and in some food sources representing environmental sources, besides direct consumer-product use (Lindström et al., 2002; Singer et al., 2002; Halden and Paull, 2005).

### DBPs

Water disinfection during the production of drinking water has been widely implemented to protect human health against waterborne diseases like cholera, typhoid, dysentery, etc (Richardson and Ternes, 2018). To this end, strong oxidants such as free chlorine, chlorine dioxide, chloramines, and ozone are used, which efficiently kill pathogens. However, disinfectants can unintentionally form DBPs by further reacting with other constituents found in waters i.e., natural organic matter, anthropogenic organic contaminants, and halide ions (chloride, bromide, iodide) (Richardson et al., 2007; Gonsior et al., 2014; Postigo and Richardson, 2014). Moreover, DBPs in pool and spa waters are formed by the reaction of disinfectants with organic matter including natural organic matter from source water and human inputs such as urine, sweat and PPCPs (Daiber et al., 2016; Jmaiff Blackstock et al., 2017). For instance, more than 100 DBPs were recently found in swimming pools and hot tubs, and organic extracts from those samples were more mutagenic than the corresponding tap water extracts (Daiber et al., 2016). Although ~700 DBP have been identified, only three classes are regularly monitored: trihalomethanes (e.g., chloroform, bromoform, bromodichloromethane, chlorodibromomethane) (**26**–**29**), haloacetic acids (e.g., chloro-, bromo-, dichloro-, dibromo-, and trichloro-acetic acid) (**30**–**34**), and oxyhalides (e.g., chlorate and bromate) (**35**–**36**) (Richardson and Ternes, 2018). People are exposed to a diverse range of DBPs by drinking, uptake through the skin upon contact, and inhalation of volatile DBPs e.g., in indoor swimming facilities (Gonsior et al., 2014; Daiber et al., 2016). Epidemiological studies have reported a relation between human ingestion of drinking water containing DBPs and increased spontaneous abortions, stillbirth, birth defects and bladder cancer in particular (Richardson et al., 2007).

## Impact

The negative impact of halogenated compounds has been known since long from a toxicological point of view. However, the role of the (gut) microbiota has not been well-incorporated into the study of interactions between environmental exposures and health outcomes (Dietert and Silbergeld, 2015).

The impact of halogenated compounds on gut microbiota has mostly been studied using rodent models (Table 1). For example, dietary exposure to 2,3,7,8-tetrachlorodibenzofuran has been shown to induce inflammation and decrease the *Firmicutes* to *Bacteroidetes* ratio in mice (Zhang L. et al., 2015). A similar decreased *Firmicutes/Bacteroidetes* ratio in mice was reported due to exposure to trichloroacetamide (**37**) (Zhang L. et al., 2015) or chlorpyrifos (**11**) (Zhao et al., 2016), and in juvenile goldfish due to exposure to pentachlorophenol (**15**) (Kan et al., 2015) (Table 1). In contrast, the *Firmicutes/Bacteroidetes* ratio increased in mice after 2,3,7,8-tetrachlorodibenzo-*p*-dioxin exposure (Lefever et al., 2016). Changes in the *Firmicutes/Bacteroidetes* ratio was first observed in obesity studies and was subsequently addressed by various studies, confirming or challenging its impact, or pointing to technical artifacts (Ley et al., 2005; Schwiertz et al., 2010; Bahl et al., 2012). Nevertheless, these ratios may in any case indicate different levels of short chain fatty acids production, pH and activity in the gut, and hence could be meaningful when methodological bias is excluded (Duncan et al., 2009; Kolmeder et al., 2015). Accordingly, treatment of mice with 2,3,7,8-tetrachlorodibenzofuran enriched *Butyrivibrio* spp., common butyrate-producing gut microbes, coupled with elevation of butyrate and propionate in feces and cecal contents (Zhang L. et al., 2015). In another study, oral exposure to polychlorinated biphenyls decreased the overall abundance of bacterial species in mice gut microbiota primarily by decreasing the levels of *Proteobacteria* (Choi et al., 2013). Interestingly, exercise attenuated alterations of mice gut microbiota composition (Choi et al., 2013). Exercise has been shown to increase beneficial metabolites, such as butyrate in the rat cecum (Matsumoto et al., 2008).

**Table 1:** Impact of halogenated compounds on gut microbiota and host (where applicable).

**Compound, concentration and duration of use**	**Compound type**	**Study model**	**Impact on gut microbiota**	**Impact on host**	**References**
2,3,7,8-tetrachlorodibenzofuran, 24 μg/kg for 5 days	POP	C57BL/6J mice (*Ahr*^+/+^) and C57BL/6J congenic mice (*Ahr^−/^*^−^)	Decreased *Firmicutes*/*Bacteroidetes* ratio; enriched *Butyrivibrio* spp. and depleted *Oscillibacter* spp. in cecal contents; production of short chain fatty acids like butyrate	Altered bile acid metabolism; significant inflammation and host metabolic disorders as a result of activation of bacterial fermentation; altered hepatic lipogenesis, gluconeogenesis and glycogenolysis in an Ahr-dependent manner	(Zhang L. et al., 2015)
Polychlorinated biphenyls (PCB) congeners (PCB153, PCB138, and PCB180) total dose of 150 μmol/kg for 2 days	POP	C57BL/6 mice	Decreased overall abundance of bacterial species; decreased levels of *Proteobacteria*; exercise attenuated PCB-induced alterations of gut microbiota composition; abundant *Lactobacillales* and depleted *Erysipelotrichaceae* bacterium C11_K211 (*Tenericutes* phylum) in the exercised group	Exercise provided protection against PCB-induced changes in the gut microbiota than sedentary mice.	Choi et al., 2013
2,3,7,8-tetrachlorodibenzo-*p*-dioxin, 0-30 μg/kg every 4 days for 28 and 92 days	POP	C57BL/6 mice	Significant increase of fourteen antimicrobial resistance genes and mobile genetic elements genes typically observed in *Enterobacteriaceae*	Increased hepatic fat accumulation; depletion of immune cell expression and populations of macrophage and dendritic cells in the intestinal lamina propria	Fader et al., 2015; Stedtfeld et al., 2017
2,3,7,8-tetrachlorodibenzo-*p*-dioxin, biweekly with a dose of 6 μg 2,3,7,8-tetrachlorodibenzo-*p*-dioxin /kg for 26 weeks	POP	CD-1 mice	Increased *Firmicutes*/*Bacteroidetes* ratio; increased *Lactobacillaceae* and *Desulfovibrionaceae*, and decreased *Prevotellaceae* and ACKM1	Liver toxicity, polydipsia (excessive thirst), polyphagia (increased appetite) and prediabetes	Lefever et al., 2016
Chlorpyrifos, 1 mg everyday for 30 days	Pesticide	Human Intestinal Microbial Ecosystem (SHIME)	Compositional change in the microbial community; increased numbers of *Enterococcus* and *Bacteroides* spp. and decreased numbers of lactobacilli and bifidobacteria		Joly et al., 2013
0.3 or 3 mg chlorpyrifos/kg bodyweight/day or for 9 weeks in rats fed a normal (NF) or high fat (HF) diet	Pesticide	Wistar rats	Reduced relative abundance of *Aerococcus, Brevundimonas*, and *Trichococcus* in NF-fed rats, and *Olsenella, Clostridium* sensu stricto 1, *Amphibacillus, Enterorhabdus*, and *Alloprevotella* in HF-fed rats	Pro-obesity phenotype in NF-fed rats; significantly reduced serum insulin, C-peptide, and amylin concentrations in NF- and HF-fed rats; no impact on serum glucose and lipid profiles	Fang et al., 2018
1 mg chlorpyrifos /kg bodyweight in corn oil once daily for 30 days	Pesticide	*Mus musculus* mice	Decreased *Firmicutes*/*Bacteroidetes* ratio; reduced relative abundance of *Lactobacillaceae* and increased relative abundance of *Bacteroidaceae*	Alterations of urine metabolites related to the metabolism of amino acids, energy, short chain fatty acids, phenyl derivatives and bile acids	Zhao et al., 2016
Chlorothalonil, 10 μg/L in a 30% sucrose solution for 6 weeks	Pesticide	Honey bees *(Apis mellifera)*	Perturbed bacterial communities but not fungal communities; reduced relative abundance of *Lactobacillaceae* and increased relative abundance of *Enterobacteriaceae* and *Caulobacteraceae*; increased putative genes for oxidative phosphorylation and declined genes for sugar metabolism and peptidase		Kakumanu et al., 2016
Pentachlorophenol, 0–100 μg/L for 28 days	Pesticide	Goldfish (*Carassius auratus*)	Decreased *Firmicutes*/*Bacteroidetes* ratio; increased relative abundance of *Bacteroides* and decreased relative abundance of *Chryseobacterium, Microbacterium, Arthrobacter* and *Legionella*	Accumulation of PCP in the fish intestinal tract in a time- and dose-dependent manner; reduced fish body weight and liver weight; antioxidant system disturbance	Kan et al., 2015
Triclosan at 0.05 mg/kg body weight, administration through milk until 28 days and afterwards through oral gavage three times a week till day 181	PPCP	Sprague Dawley rats	Decreased *Firmicutes* /*Bacteroidetes* ratio; increased *Deltaproteobacteria* and *Lactobacillus*, increased *Lachnospiraceae*	Reduction in the bodyweight in adolescent rats	Hu et al., 2016
Triclosan in water solution (2 mg/L) for 13 weeks	PPCP	C57BL/6 mice	Decreased alpha diversity; depletion of *Turicibacteraceae, Christensenellaceae* and *Clostridiales*; enrichment of gut bacterial genes related to triclosan resistance, stress response, antibiotic resistance and heavy metal resistance		Gao et al., 2017
Triclocarbon, supplemented in feed (0.1% w/w) for 12 days	PPCP	Sprague Dawley rats	Significantly reduced phylogenetic diversity of gut among exposed dams and neonates during gestation and lactation; dominance of *Enterobacteriaceae*		Kennedy et al., 2016
Commercially available wash products either containing or not containing triclosan for 1 year	PPCP	Humans: 39 pairs of mothers and babies	No global reconstruction or loss of microbial diversity of either infant or maternal gut microbiotas; broadly antibiotic-resistant species from the phylum *Proteobacteria* were enriched in stool samples from mothers		Ribado et al., 2017
Triclosan-containing PPCP (4 months) and non-triclosan-containing PPCP (4 months)	PPCP	Humans (16 persons)	No differences in microbiota composition, species richness and overall diversity of the stool, molar, or incisor	Higher urinary concentrations of triclosan in all volunteers during the triclosan period; no differences in metabolic or endocrine markers, or weight	Poole et al., 2016
Chloroacetate, bromoacetate, dichloroacetate, dibromoacetate, trichloroacetate, tribromoacetate, or bromochloroacetate; 1 gm/ml of each compound in selective gorwth media	DBP	Incubations of CDF rat cecal microbiota	Toxic impacts on cecal microbiota especially to the enterococci; increased activities for β-glucuronidase, β-galactosidase, β-glucosidase, azoreductase, nitroreductase, dechlorinase, and dehydrochlorinase that can affect the biotransformation of co-exposed compounds		Nelson et al., 2001
Trichloroacetamide, 50, 500 and 5000 μg/l for 90 days	DBP	*Mus musculus* mice	Decreased *Firmicutes*/*Bacteroidetes* ratio with an increase in the concentration of trichloroacetamide; Increased relative abundance of *Bacteroidaceae, Porphyromonadaceae, Sphingobacteriaceae, Aerococcaceae*, and *Erysipelotrichaceae* and decreaseed relative abundance of *Bacillaceae, Heliobacteriaceae, Syntrophomonadaceae*	Disruption of the host metabolism, weight loss, altered choline metabolites in urine samples; decreased urine tyrosine and intestinal lesions; disordered amino acid and lipid metabolism, alterations in the serum metabolome, including altered choline, trimethylamino oxide, as well as hepatotoxicity and cytotoxicity	(Zhang et al., 2013; Zhang Y. et al., 2015)

Organohalogen POPs accumulate in adipose tissue in humans because of their lipophilicity. These POPs have been shown to bind to aryl hydrocarbon receptor (AHR), that is a transcription factor involved in the regulation of biological responses to planar aromatic (aryl) hydrocarbons (Arsenescu et al., 2008). This xenobiotic sensor modulates the activity of immune and nonimmune cells in the gut, and may represent an important link between the environment and immune system perturbations (Monteleone et al., 2012). In line with this, *ahr*-knockout (*Ahr*^−/^^−^) mice did not show large shifts in gut microbial composition in response to 2,3,7,8-tetrachlorodibenzofuran exposure (Zhang L. et al., 2015). Modulation of gut microbiota by AHR was proposed to play an important role in the induction of obesity by chronic POP exposure (Myre and Imbeault, 2014). For instance, exposure to polychlorinated biphenyls has been shown to impair glucose homeostasis in mice (Baker et al., 2013). Furthermore, higher body burden of dioxins, and polychlorinated biphenyls was reported in obese people as opposed to lean people (Kim et al., 2011). A comprehensive literature review on the impact of chlorinated POPs in humans showed that rather than a few individual POPs, background exposure to low-dose POP mixtures may promote type 2 diabetes and obesity (Lee et al., 2014). In another survey, evaluation of 72 epidemiological studies revealed the strongest positive correlation of diabetes with organochlorine than non-organochlorine POPs (Taylor et al., 2013).

In a recent *in vitro* study, the Simulator of the Human Intestinal Microbial Ecosystem (SHIME) model was inoculated with feces from healthy humans, and subsequently exposed to chronic and low-doses of the insecticide chlorpyrifos (**11**). This induced major changes in the microbial community, in particular, increased numbers of *Enterococcus* and *Bacteroides* spp., and decreased numbers of lactobacilli and bifidobacteria, the latter including probiotics commonly associated with health benefits (Joly et al., 2013). Compositional shifts in intestinal bacterial community structure and distortion of their metabolic functions were similarly reported due to exposure of rats to chlorpyrifos (Zhao et al., 2016; Fang et al., 2018) (Table 1). In another study, oral exposure of bees to the organochlorine fungicide chlorothalonil (**16**) induced microbial changes, increased putative genes for oxidative phosphorylation and declined sugar metabolism and peptidase potential (Kakumanu et al., 2016). In contrast to these reports, early-life exposure to the estimated environmental concentration of atrazine (**12**) (200 μg/L) did not affect gut bacterial diversity or community composition of tadpoles (*in vivo* or *in vitro*) or adult frogs (Knutie et al., 2018). Discrepancies may arise due to dose, timing, route of exposure, host type and metabolism, and the applied chemical differences.

Besides the known impact of (halogenated) antibiotics in inducing major but partly reversible changes in gut microbiota composition (Dethlefsen and Relman, 2011; Vrieze et al., 2014), an increasing number of studies have shown microbiota perturbations by non-antibiotic biocides. For example, a recent comprehensive screening of more than 1,000 non-antibiotic drugs against 40 representative gut bacterial strains showed that 24% of the drugs with human targets inhibited the growth of at least one strain *in vitro* (Maier et al., 2018). Some intensively used organohalogen drugs such as thyroxine (L) (**24**), a medication used to treat thyroid hormone deficiency, and the anti-inflammatory diclofenac (**25**) were among the tested compounds (Maier et al., 2018). In another study, adolescent rats receiving triclosan orally at levels comparable to human exposures showed lower gut microbiota diversity and more noticeable compositional changes, whereas these differences were diminished in adult rats (Hu et al., 2016). Moreover, triclosan exposure was reported to reduce alpha diversity in the gut microbiota of rats (Kennedy et al., 2016; Gao et al., 2017) (Table 1). In contrast, triclosan exposure experiments in humans have not shown major perturbations in the gut and oral microbiota (Poole et al., 2016; Ribado et al., 2017) (Table 1). Since humans experience much lower triclosan exposures in products such as soap and toothpaste that are rinsed off immediately, the impacts observed in high-dose and acute animal exposures might not be observed in humans. However, a positive correlation was reported between the exposure to triclosan and the occurrence of *Staphylococcus aureus* as an opportunistic pathogen in the human nasal microbiota (Syed et al., 2014). There are also concerns regarding contribution of non-antibiotic antimicrobials to antibiotic resistance due to cross-resistance (Hartmann et al., 2016; Maier et al., 2018). For example, prolonged exposure to triclosan was associated with developing resistance and cross-resistance to ampicillin and/or ciprofloxacin in *S. aureus* and *Escherichia coli* (Wesgate et al., 2016). Exposure of a susceptible *Pseudomonas aeruginosa* strain to triclosan has been shown to select multidrug-resistance mediated by multidrug efflux pumps (Chuanchuen et al., 2001). Similarly, abundance of several multidrug-resistance efflux pump genes was reported to significantly increase after triclosan exposure (Gao et al., 2017). Recent studies on the mouse gut indicated the selective pressure of 2,3,7,8-tetrachlorodibenzo-*p*-dioxin in promoting blooms of *Enterobacteriaceae*, that harbor antimicrobial resistance genes (Stedtfeld et al., 2017) (Table 1). Similar increased levels of *Enterobacteriaceae* were reported in: (i) the gut of honey bees exposed to the organochlorine fungicide chlorothalonil (**16**) (Kakumanu et al., 2016), and (ii) the human gut due to gastrointestinal infection (Lupp et al., 2007) and antibiotic therapies (Sekirov et al., 2008). Finally, DBPs have the potential to select for antibiotic resistance (Li et al., 2016; Zhang et al., 2016). These reports indicate similar impacts despite different types of stressors and hosts.

## Fate

Extensive studies have been performed to understand the fate of xenobiotics by oxidative and conjugative enzymes in the liver (Zanger et al., 2008; Zanger and Schwab, 2013). Among the hepatic enzymes, cytochrome P-450s are the major oxidative enzymes for transformation of (halogenated) lipophilic xenobiotics and drugs e.g., PCDDs (Hu and Bunce, 1999) and diclofenac (Leemann et al., 1993). In contrast, much less is known about the dehalogenation and/or degradation of halogenated xenobiotics by intestinal microbiota that employ hydrolytic and reductive mechanisms (Sousa et al., 2008).

### Cultivation-dependent view

**Figure 3:**
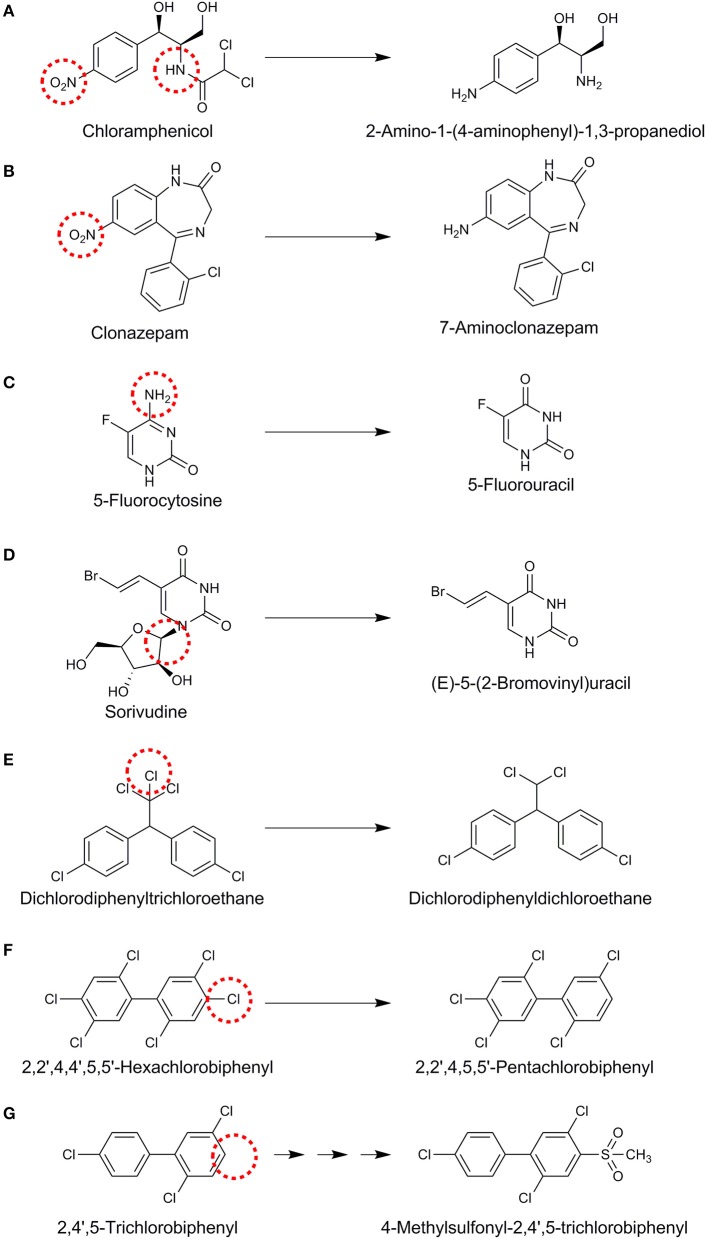
Proposed reactions for different organohalogens **(A–G)** mediated by gut microbiota. Reaction sites are indicated with red dashed circles.

Understanding of the fate of halogenated xenobiotics using cultivation-based studies has been derived from the exposure of the intestinal contents or specific microbial isolates of intestinal origin. For example, incubation of chloramphenicol, that contains a nitrobenzene group and an amide of dichloroacetic acid, with human fecal bacteria led to the hydrolysis of the amide linkage and reduction of the nitro group to an amine on the aromatic ring (Figure 3A) (Holt, 1967). Nitroreductases reducing nitro (–NO_2_) functional groups to the corresponding amines are an important group of enzymes identified for the gut microbial xenobiotic metabolism (Rickert et al., 1981; Claus et al., 2016). Another example of nitroreductive metabolism of organohalogens was shown for clonazepam, a medication used to prevent and treat seizures and panic disorder, that was converted to 7-aminoclonazepam by rat intestinal lumen microbiota (Figure 3B) (Elmer and Remmel, 1984). In contrast to such amine group formation, amine group removal by human intestinal microbiota was reported for the anti-fungal 5-fluorocytosine (Figure 3C) (Harris et al., 1986; Vermes et al., 2003). Susceptible fungi contain a cytosine deaminase which converts 5-fluorocytosine to 5-fluorouracil (Figure 3C). The latter is further metabolized to 5-fluorodeoxyuridylic acid, an inhibitor of thymidylate synthetase and subsequently DNA synthesis (Vermes et al., 2000). Although human host cells lack the deaminase enzyme, 5-fluorocytosine conversion to 5-fluorouracil by the human intestinal microbiota plays an important role in the development of hematologic and gastrointestinal toxicity (Harris et al., 1986). Co-administration of 5-fluorocytosine with the antiviral drug sorivudine led to 18 acute deaths due to an unknown lethal gut microbial metabolism (Okuda et al., 1998). Further research has revealed that intestinal *Bacteroides* species, namely *Bacteroides vulgatus, B. thetaiotaomicron, B. fragilis, B. uniformis*, and *B. eggerthii* can convert sorivudine to (E)-5-(2-bromovinyl)uracil (Nakayama et al., 1997) (Figure 3D), whereas the latter was barely detected in the plasma of germ-free rats (Ashida et al., 1993). A key liver enzyme that regulates the systemic 5-fluorocytosine level is subsequently inactivated by (E)-5-(2-bromovinyl)uracil, leading to toxic levels of 5-fluorocytosine and death in rats and humans (Okuda et al., 1998). This is an important example of the role of gut microbiota in toxification processes.

Reductive dehalogenation of dichlorodiphenyltrichloroethane to dichlorodiphenyldichloroethane (Figure 3E) has been shown in anoxic incubations of the strictly anaerobic human intestinal bacterium *Eubacterium limosum* (Yim et al., 2008) and rat intestinal microbiota (Mendel and Walton, 1966). However, it is not known if this is a bioactivation or detoxification mechanism as dichlorodiphenyldichloroethane is still an endocrine disruptor (Claus et al., 2016). Reductive dehalogenation can be mediated co-metabolically by vitamin B_12_ (cobalamin) that is synthesized by some human gut microbes (Degnan et al., 2014). In contrast, metabolic reductive dehalogenation is mediated by specific bacterial groups that can use organohalogens as their terminal electron acceptors (Smidt and de Vos, 2004; Atashgahi et al., 2016). A co-culture of *Clostridium perfringens* and *C. beijerinckii* was also shown to reductively dehalogenate hexachlorobiphenyl to pentachlorobiphenyl (Figure 3F) and tetrachlorobiphenyl to trichlorobiphenyl (De et al., 2006). Gut microbiota was also shown to be involved in generation of methylsulfone (MeSO_2_) metabolites from polychlorinated biphenyls (PCBs) through a series of reactions in combination with the host cells (Figure 3G) (Bakke et al., 1982; Brandt et al., 1982). MeSO_2_–PCBs can bind to specific proteins and accumulate in the lipophylic tissues with adverse effects (Shigematsu et al., 1978). Exposure to 2,3,7,8-tetrachlorodibenzofuran has been shown to enhance the level of *Flavobacteria* in the gut of mice (Zhang L. et al., 2015). These bacteria are reported to possess glutathione-dependent reductive dehalogenase activity (Xun et al., 1992), although 2,3,7,8-tetrachlorodibenzofuran dehalogenation by the gut microbiota has not been shown yet.

Intestinal lactobacilli have been reported to degrade organohalogens *in vitro*. For instance, *Lactobacillus lactis, L. fermentum, L. plantarum, E. coli*, and *Enterococcus faecalis* were tested for chlorpyrifos degradation potential. The results indicated that besides *E. coli, L. lactis* and *L. fermentum* could grow in the presence of over 1.5 mg/mL chlorpyrifos (Harishankar et al., 2013). Similarly, four lactic acid bacteria isolated from kimchi fermentation in the presence of 200 mg/L chlorpyrifos were reported to use this pesticide as the sole source of carbon and phosphorus (Cho et al., 2009). Moreover, lactic acid bacteria seeded to skimmed milk were shown to degrade the insecticide trichlorfon (**17**) (Zhao and Wang, 2012). However, the actual impact of lactobacilli on organohalogen fate in the gut is not known, especially since this bacterial group seems particularly sensitive to organohalogen exposure (Table 1).

### Cultivation-independent view

As a complementary approach to cultivation, metagenomic approaches can also be used to infer xenobiotic metabolism potential by the gut microbiota (Haiser and Turnbaugh, 2013; Spanogiannopoulos et al., 2016). Due to environmental persistence and toxicity, great attention has been given to understand microbial transformation of halogenated xenobiotics in environmental studies with the end goal of bioremediation. Indeed a variety of microbial dehalogenation mechanisms have been described that can remove halogens from organic compounds by oxidation, reduction and substitution mechanisms that are employed in co-metabolic and/or energy-yielding modes (van Pée, 1996; Fetzner, 1998; Janssen et al., 2001; Smidt and de Vos, 2004).

**Figure 4:**
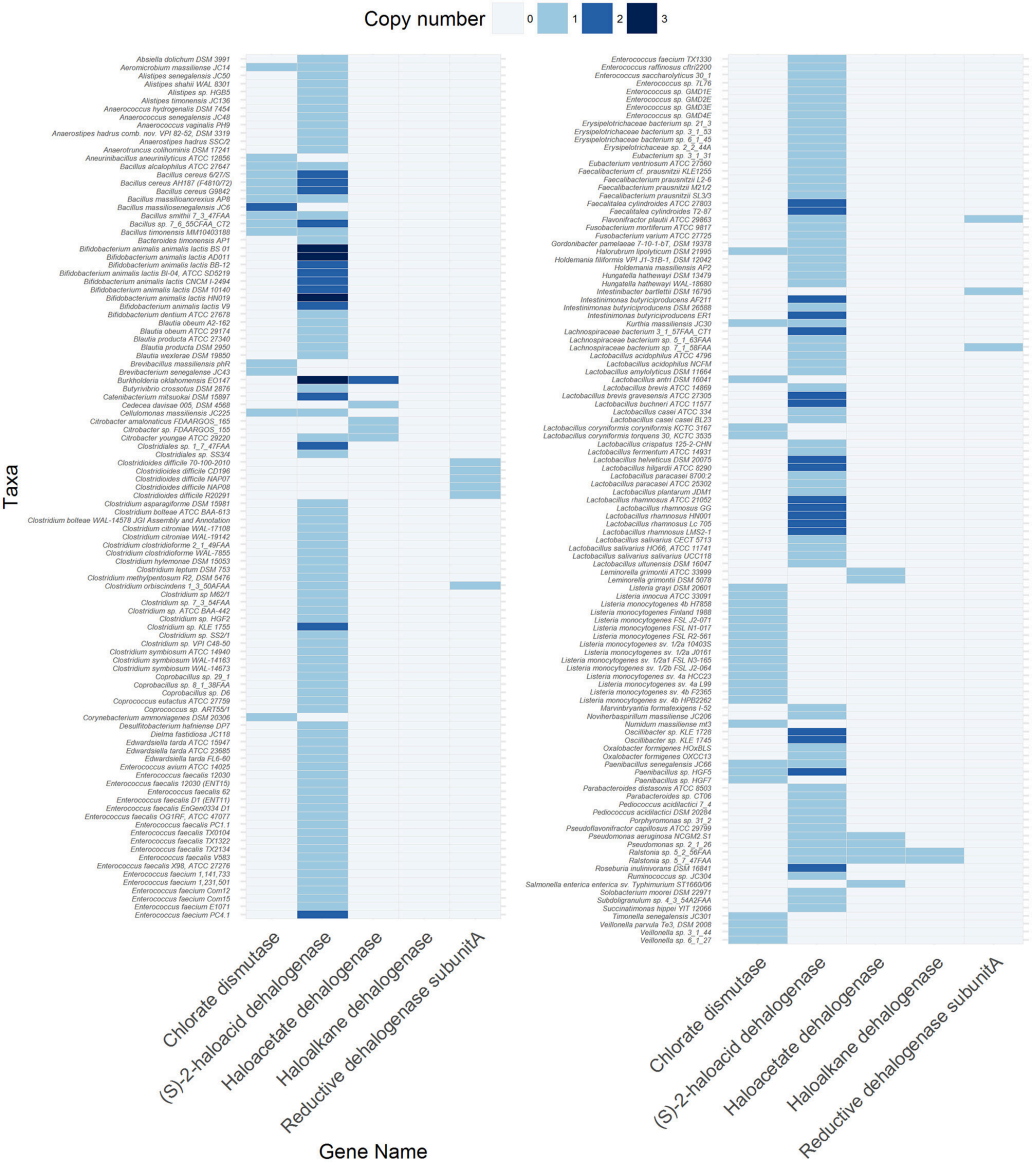
Genes encoding different dehalogenases found in 216 out of 670 bacterial and archaeal genomes of gut origin. The code to reproduce the figure is available at https://github.com/mibwurrepo/Atashgahi-et-al.-XenobioticReview2018.

Although a metagenomic view of xenobiotic metabolism in the gut has been provided (Haiser and Turnbaugh, 2013; Spanogiannopoulos et al., 2016), specific information on the prevalence and diversity of known dehalogenase-encoding genes in the gut metagenome is lacking. Therefore, we surveyed the Joint Genome Institute Integrated Microbial Genomes & Microbiome System (JGI-IMG/MER) database for the occurrence of different dehalogenating gene classes (Table S1) in 670 bacterial and archaeal genomes from fecal origin (Table S2), 254 metagenomes obtained from human fecal samples (Table S3) and 86 metagenomes obtained from gut/rumen fluid/fecal samples of animals (Table S4). The metadata and criteria used for the selection of genomes and metagenomes are listed in Tables S2–4. The codes used for the analysis and visualization are provided in supplementary information. The results showed that at least one dehalogenating gene was present in 32.2% of the bacterial and archaeal genomes, and in 61 and 75.6% of the metagenomes derived from human and non-human origins, respectively (Tables S5–S7). Among these, five types of genes were found in the bacterial genomes (Figure 4, Table S5) and human metagenomes (Table S6), and six types of genes in non-human metagenomes (Table S7). Four types of genes were shared among the three datasets and were predicted to code for (S)-2-haloacid dehalogenase (EC:3.8.1.2), haloacetate dehalogenase (EC:3.8.1.3), haloalkane dehalogenase (EC:3.8.1.5), and reductive dehalogenase (EC:1.21.99.5). In contrast, a canonical gene for chlorate dismutase (EC: 1.13.11.49) was not found in the human metagenomes whereas that for atrazine chlorohydrolase (EC:3.8.1.8) was absent from the bacterial genomes. Atrazine chlorohydrolase catalyzes the conversion of the herbicide atrazine to hydroxyatrazine, the first step in the atrazine degradation pathway (Mandelbaum et al., 1995).

A gene for (S)-2-haloacid dehalogenase was the most abundant in both the genome (214 genes) and metagenome (2612 genes in human and 4549 genes in non-human) datasets indicating exposure of gut microbiota to haloacids that are common DBPs (Richardson et al., 2007). Many of the known core gut genera, such as *Faecalibacterium, Blautia, Roseburia, Alistipes, Eubacterium* (Shetty et al., 2017) harbor a (S)-2-haloacid dehalogenase gene in their genome (Figure 4, Table S5). The encoded enzyme belongs to the family of hydrolases, acting specifically on halide bonds in α-substituted haloacids (Janssen et al., 2001). Interestingly, a (S)-2-haloacid dehalogenase encoding gene co-occurred with that of chlorite dismutase in 14 microbial genomes (Figure 4, Table S5). Chlorite dismutase mediates the last step in chlorate reduction splitting chlorite to chloride and oxygen (Liebensteiner et al., 2016). An environmental bacterium, *Pseudomonas chloritidismutans* AW-1^T^, which similarly harbored these two genes, was recently shown to concurrently degrade haloacids and chlorate as the electron donor and acceptor, respectively (Peng et al., 2017). This is an interesting finding considering that haloacids and chlorate are common DBPs in drinking water resources (Richardson et al., 2007) that might be degraded by the gut microbes harboring these genes. Further, the oxygen produced from chlorite dismutation can be used for degradation of haloacids (Peng et al., 2017) or other organic compounds (Oosterkamp et al., 2013; Atashgahi et al., 2018b) in an “intra-aerobic” pathway. If functional, this can be an important metabolism in the gut environment where oxygen is largely unavailable to serve as a terminal electron acceptor.

The well-studied reductive dehalogenase genes were more abundant in the non-human than in the human metagenomes (Table S7). Interestingly, of the 59 reductive dehalogenase genes found in the non-human metagenomes, 53 were from the rumen content of sheep, goat and cow. This implies that the rumen content of ruminant farm animals is an appropriate environment for the reductive dehalogenation metabolism that represents useful but largely unexplored sources for future enrichment/isolation of organohalide-respiring bacteria. These bacteria reductively dehalogenate organohalogens by replacing the halogen substitutes with hydrogen in a process known as organohalide respiration (Smidt and de Vos, 2004; Atashgahi et al., 2016). This metabolism usually reduces the toxicity of organohalogens and makes the otherwise chemically locked organohalogens available to other microbial metabolisms such as fermentative and aerobic degradation. Organohalide-respiring bacteria have only been found in pristine and contaminated environments impacted by natural or anthropogenic organohalogens (Atashgahi et al., 2018a). The activity of these microbes in host-associated ecosystems and their potential impacts on the organohalogen fate especially in the gut remains largely unknown. There is only one study showing reductive dehalogenation of polychlorinated biphenyls by the co-culture of *Clostridium perfringens* and *C. beijerinckii* as prominent species in the human gut (De et al., 2006) (Figure 3F). Interestingly, we found six reductive dehalogenase genes in the genomes of *Clostridia* (Figure 4, Table S5). This may point to an unrecognized reductive dehalogenation potential in the gut microbiota. Of interest, the gut isolate strain DP7, belonging to the genus *Desulfitobacterium* (*Clostridia*) that is known for its active organohalogen respiration metabolism (Kruse et al., 2017), was reported to lack reductive dehalogenation activity (van de Pas et al., 2001) and the corresponding genes (Kruse et al., 2017).

## Conclusions and outlook

The toxicant-microbiota interaction has emerged in recent years as one of the novel concepts from the intensive research on the human microbiome. The gut microbiota constitutes a critical zone for xenobiotic (de)toxification and sequestration at the interphase between the external environment and our mucosal epithelial cells. An important class of toxicants are halogenated compounds from anthropogenic and natural sources that come into contact with the human gut and other body parts mainly by ingestion of, or exposure to contaminated food and water. Although canonical toxicological approaches using short-term high-dose exposure experiments are informative about the toxicity and impact of halogenated compounds, they do not represent scenarios of chronic exposure to low-level xenobiotic cocktails throughout life. The ingested concentrations of the halogenated xenobiotics in food and water resources are in most cases below the regulatory thresholds. However, little is known about the physiological impact, reactivity, bioaccumulation in food chains, additive/cumulative toxicity of these emerging contaminants, and their (bio)transformation products. For example, even at trace concentrations, mixtures of biocides, antibiotics, and heavy metals have the potential to contribute to the emergence, maintenance and transmission of antibiotic-resistant and disease-causing bacteria (Gullberg et al., 2014; Pal et al., 2015). Therefore, future studies are necessary to reveal the impact of halogenated (micro)pollutants on the gut microbial community membership, gene expression, physiology, metabolite profile, antibiotic resistance genes, and also parallel impacts on the host. Long-term incubations of the gut contents/isolates with environmentally relevant doses and diversity of halogenated compounds, in combination with other micropollutants, should aid in understanding the actual consequences of chronic low-dose exposures.

Even less information is available on the specific microorganisms responsible for halogenated xenobiotic metabolism, the molecular mechanisms and biotransformation pathways involved that can either diminish or enhance the toxicity. The metagenomic approach described here showed that genes involved in dehalogenation are widespread among gut bacteria, and this may impact flux, toxicity, bioavailability and fate of halogenated compounds. Future cultivation and omics experiments are necessary to test the actual metabolism of the halogenated compounds by the gut microbiota. To this end, we can immensely benefit from the wealth of knowledge gained about the metabolism of halogenated xenobiotic compounds in terrestrial and aquatic environments (Janssen et al., 2001; Smidt and de Vos, 2004; Atashgahi et al., 2018a) and high-throughput cultivation of the gut microbiota (Ingham et al., 2007; Lagier et al., 2016). These approaches should be coupled with untargeted metabolomics using high-resolution mass spectroscopy to identify xenobiotics and biotransformation products. Untargeted metabolomics has the potential to aid in determination of pathways and mechanisms of action (Warth et al., 2017).

Given the immense potential of gut microbiota to alter the chemical structure and bioactivity of xenobiotics with beneficial (Shin et al., 2013) or severely detrimental impacts (Okuda et al., 1998), assessments of xenobiotic metabolism should be an integral part of designing drugs and chemicals such as PPCPs and pesticides, informing toxicology risk assessment, improving nutrition, and guiding personalized medicine.

## Author contributions

SA, HS and WMdV have designed the study, SAS has performed the metagenomic analysis and all authors wrote the manuscript.

### Conflict of interest statement

The authors declare that the research was conducted in the absence of any commercial or financial relationships that could be construed as a potential conflict of interest.
