# Supplementary material for: Flux, Impact, and Fate of Halogenated Xenobiotic Compounds in the Gut
Source: Front Physiol. 2018 Jul 10;9:888. doi: 10.3389/fphys.2018.00888 (PMC6048469; doi:10.3389/fphys.2018.00888)
Supplement: Supplementary file 2 [file Data_Sheet_2.DOCX]

**Flux, impact and fate of halogenated xenobiotic compounds in the gut**

Siavash Atashgahi^1^, Sudarshan A. Shetty^1^, Hauke Smidt^1^, Willem M. de Vos^1,2*^

^1^ Laboratory of Microbiology, Wageningen University & Research, Stippeneng 4, 6708 WE Wageningen, the Netherlands

^2^ Research Programme Unit Immunobiology, Department of Bacteriology and Immunology, Helsinki University, Helsinki, Finland

**Correspondence:** willem.devos@wur.nl

This is the file with codes for analyses done in the review article by Atashgahi et al (2018) entitled: Flux, impact and fate of halogenated xenobiotic compounds in the gut. *Front. Physiol. - Gastrointestinal Sciences*.
The required files and Rmarkdown files can be found at the Github repository: [mibwurrepo/Atashgahi-et-al.-XenobioticReview2018](https://github.com/mibwurrepo/Atashgahi-et-al.-XenobioticReview2018).

Please in install the following packages and load them for analysis.

library(ggplot2)
library(tidyverse)
library(knitr)
library(plyr)
library(RColorBrewer)

The genome cart i.e. genomecart_export.txt file in the Genomes folder can be used to upload to IMG/M ER website. This will select all genomes used here and add them to genome cart.

Since IMG has a limit of 500 genomes for searching functional profiles, the data was obtained in two sets. First was 446 genomes and the remaining 230 in the second set. These were then merged in one file genome_results_EC_hits.txt in folder named Genomes.
Read the output of IMG-MER into R for visualisation.

# read the results from IMG database downloaded on 20-apr-2018
genome_counts <- read.table("./Genomes/genome_results_EC_hits.txt",
 sep = "\t", row.names = 1,
 stringsAsFactors = FALSE, header = T)


dim(genome_counts)

## [1] 670 29

colnames(genome_counts)

## [1] "Domain" "EC.1.13.11.49" "EC.1.21.99.1" "EC.1.21.99.2"
## [5] "EC.1.21.99.3" "EC.1.21.99.4" "EC.1.97.1.1" "EC.1.97.1.10"
## [9] "EC.1.97.1.11" "EC.1.97.1.8" "EC.3.8.1.." "EC.3.8.1.1"
## [13] "EC.3.8.1.10" "EC.3.8.1.11" "EC.3.8.1.2" "EC.3.8.1.3"
## [17] "EC.3.8.1.4" "EC.3.8.1.5" "EC.3.8.1.6" "EC.3.8.1.7"
## [21] "EC.3.8.1.8" "EC.3.8.1.9" "EC.4.5.1.." "EC.4.5.1.1"
## [25] "EC.4.5.1.2" "EC.4.5.1.3" "EC.4.5.1.4" "EC.4.5.1.5"
## [29] "pfam13486"

# Remove the first column as the information is not useful for this project.
genome_counts.2 <- genome_counts[,-1]

# Add a new column to count total genes detected in each genome

genome_counts.2$Total <- rowSums(genome_counts.2)
dim(genome_counts.2)

## [1] 670 29

# interactive table

#kable(genome_counts.2)

# now add a coulm with genome names
genome_counts.2$Taxa <- rownames(genome_counts.2)

barplot(genome_counts.2$Total)


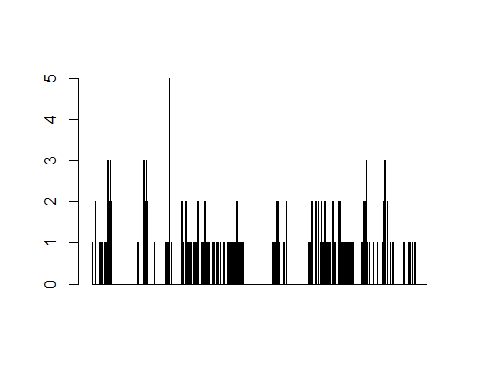


# remove genomes with no hits
genome_counts.3 <- genome_counts.2[, -which(numcolwise(sum)(genome_counts.2) == 0)]

# check which are not present
setdiff(colnames(genome_counts.2), colnames(genome_counts.3))

## [1] "EC.1.21.99.1" "EC.1.21.99.2" "EC.1.21.99.3" "EC.1.21.99.4"
## [5] "EC.1.97.1.1" "EC.1.97.1.10" "EC.1.97.1.11" "EC.1.97.1.8"
## [9] "EC.3.8.1.." "EC.3.8.1.1" "EC.3.8.1.10" "EC.3.8.1.11"
## [13] "EC.3.8.1.4" "EC.3.8.1.6" "EC.3.8.1.7" "EC.3.8.1.8"
## [17] "EC.3.8.1.9" "EC.4.5.1.." "EC.4.5.1.1" "EC.4.5.1.2"
## [21] "EC.4.5.1.3" "EC.4.5.1.4" "EC.4.5.1.5"

genome_counts.4 <- subset(genome_counts.3, Total != 0)

genome_counts.4$Total <- NULL
str(genome_counts.4)

## 'data.frame': 216 obs. of 6 variables:
## $ EC.1.13.11.49: int 0 1 0 0 0 0 0 0 0 0 ...
## $ EC.3.8.1.2 : int 1 1 1 1 1 1 1 1 1 1 ...
## $ EC.3.8.1.3 : int 0 0 0 0 0 0 0 0 0 0 ...
## $ EC.3.8.1.5 : int 0 0 0 0 0 0 0 0 0 0 ...
## $ pfam13486 : int 0 0 0 0 0 0 0 0 0 0 ...
## $ Taxa : chr "Absiella dolichum DSM 3991 " "Aeromicrobium massiliense JC14 " "Alistipes senegalensis JC50 " "Alistipes shahii WAL 8301 " ...

nrow(genome_counts)

## [1] 670

nrow(genome_counts.4)

## [1] 216

# 216 genomes out of 670 had at least one of the following E.C
dim(genome_counts.4)

## [1] 216 6

# rename EC to names
colnames(genome_counts.4)

## [1] "EC.1.13.11.49" "EC.3.8.1.2" "EC.3.8.1.3" "EC.3.8.1.5"
## [5] "pfam13486" "Taxa"

# [1] EC.1.13.11.49 EC.3.8.1.2 EC.3.8.1.3 EC.3.8.1.5 pfam13486
# "Chlorite O(2)-lyase", "(S)-2-haloacid dehalogenase", "Haloacetate dehalogenase", "Haloalkane dehalogenase", "Reductive dehalogenase subunitA"
colnames(genome_counts.4) <- c("Chlorite O(2)-lyase", "(S)-2-haloacid dehalogenase", "Haloacetate dehalogenase", "Haloalkane dehalogenase", "Reductive dehalogenase subunitA", "Taxa")


## save file for supplementary table
#write.csv(genome_counts.4, "./676_genomes/Supp_Genome_hists_216.csv", row.names = T)

df_1 <- genome_counts.4[1:105,]
df_2 <- genome_counts.4[106:216,]
df_1.melt <- reshape2::melt(df_1)

## Using Taxa as id variables

df_2.melt <- reshape2::melt(df_2)

## Using Taxa as id variables

colnames(df_1.melt) <- c("Taxa", "variable", "value")
colnames(df_2.melt) <- c("Taxa", "variable", "value")
# df1
#unique(df_1.melt$Taxa)


p1 <- ggplot(df_1.melt, aes(reorder(Taxa, desc(Taxa)),
 variable,
 fill = as.factor(value))) + geom_tile(color = "grey90") + coord_flip() +
 scale_fill_manual("Copy number", values = alpha(c(
 "#f4f8fc", "#9ecae1", "#225ea8", "#001d54"
 ), 1)) + theme_minimal() +
 theme(
 axis.text.x = element_text(
 angle = 45,
 vjust = 1,
 hjust = 1,
 size = 10
 ),
 axis.text.y = element_text(size = 4, face = "italic"),
 legend.text = element_text(size = 6)
 ) + ylab("Gene Name") + xlab("Taxa")
p1 <- p1 + scale_y_discrete(
 labels = c(
 "Chlorate dismutase",
 "(S)-2-haloacid dehalogenase",
 "Haloacetate dehalogenase",
 "Haloalkane dehalogenase",
 "Reductive dehalogenase subunitA"
 )
 )
p1

p2 <- ggplot(df_2.melt, aes(reorder(Taxa, desc(Taxa)),
 variable, fill = as.factor(value))) +
 geom_tile(color = "grey90") + coord_flip() +
 scale_fill_manual("Copy number", values = alpha(c(
 "#f4f8fc", "#9ecae1", "#225ea8", "#001d54"
 ), 1)) + theme_minimal() +

 theme(
 axis.text.x = element_text(
 angle = 45,
 vjust = 1,
 hjust = 1,
 size = 10
 ),
 axis.text.y = element_text(size = 4, face = "italic"),
 legend.text = element_text(size = 6)
 ) + ylab("Gene Name")

p2 <- p2 + scale_y_discrete(
 labels = c(
 "Chlorate dismutase",
 "(S)-2-haloacid dehalogenase",
 "Haloacetate dehalogenase",
 "Haloalkane dehalogenase",
 "Reductive dehalogenase subunitA"
 )
 )
p2 <- p2 + theme(axis.title.y=element_blank())

p3 <- ggpubr::ggarrange(p1, p2, ncol = 2,common.legend = TRUE)

# Save the figures
#ggsave("./Genomes/Figure 4.pdf", height = 10, width = 10)
#ggsave("./Genomes/Figure 4.tiff", height = 9, width = 8, dpi = 300)

Similar to genomes, the list of human gut metagenomes can be found in metagenomes_export_254.txt in the Human_gut_metagenomes folder.

## Metagenome search

library(dplyr)

counts.met <- read.table("./Human_gut_metagenomes/metagenomes_EC_counts_254.txt", sep = "\t", row.names = 1, stringsAsFactors = FALSE, header = T)
counts.met <- counts.met[,-1]
#head(counts.met)
counts.met$Total <- rowSums(counts.met)
counts.met$ID <- paste0( "Sample-", seq.int(nrow(counts.met)))

#unique(counts.met$Total)


frm <- counts.met %>%
 mutate(Total = as.numeric(Total)) %>%
 mutate(TotalGRP = case_when(
 Total < 10 ~ 'less than 10',
 between(Total, 10, 30) ~ 'between 10 to 30',
 between(Total, 29, 50) ~ 'between 29 to 50',
 Total > 50 ~ 'more than 50')
 )

#frm$TotalGRP


counts.met2 <- frm[, -which(numcolwise(sum)(frm) == 0)]
setdiff(colnames(counts.met), colnames(counts.met2))

## [1] "EC.1.13.11.49" "EC.1.21.99.1" "EC.1.21.99.2" "EC.1.21.99.3"
## [5] "EC.1.21.99.4" "EC.1.97.1.1" "EC.1.97.1.10" "EC.1.97.1.11"
## [9] "EC.1.97.1.8" "EC.3.8.1.." "EC.3.8.1.1" "EC.3.8.1.10"
## [13] "EC.3.8.1.11" "EC.3.8.1.4" "EC.3.8.1.6" "EC.3.8.1.7"
## [17] "EC.3.8.1.9" "EC.4.5.1.." "EC.4.5.1.1" "EC.4.5.1.2"
## [21] "EC.4.5.1.3" "EC.4.5.1.4" "EC.4.5.1.5"

ncol(counts.met)

## [1] 30

colnames(counts.met2)

## [1] "EC.3.8.1.2" "EC.3.8.1.3" "EC.3.8.1.5" "EC.3.8.1.8" "pfam13486"
## [6] "Total" "ID" "TotalGRP"

counts.met2 <- counts.met2[ ,-6]
colnames(counts.met2) <- c("(S)-2-haloacid dehalogenase", "Haloacetate dehalogenase", "Haloalkane dehalogenase", "Atrazine chlorohydrolase", "Reductive dehalogenase subunitA", "ID", "TotalGRP")
counts.met2.df <- reshape2::melt(counts.met2)

## Using ID, TotalGRP as id variables

#head(counts.met2.df)
colnames(counts.met2.df) <- c("Sample", "TotalGRP","Gene", "Counts")

#head(counts.met2.df)

unique(counts.met2.df$pfam13486)

## NULL

p1 <- ggplot(counts.met2.df,
 aes(Sample, Gene,
 fill = TotalGRP)) +
 geom_tile(color = "grey90") +
 scale_fill_manual("Copy number", values = alpha(c(
 "#f4f8fc", "#9ecae1", "#225ea8", "#001d54"
 ), 1)) + theme_bw() +
 theme(
 axis.title.x = element_blank(),
 axis.text.x = element_blank(),
 axis.ticks.x = element_blank()
 ) + ylab("Gene Name")


#ggsave("./Human_gut_metagenomes/Dehalogenase_related_metagenome.pdf", height = 3, width = 10)


#ggsave("./human_gut_metagenomes/Dehalogenase_related_metagenome.tiff", height = 3, width = 10, dpi = 300)

For queries regarding this analysis, contact Sudarshan Shetty at [sudarshanshetty9@gmail.com](mailto:sudarshanshetty9@gmail.com)
